# Identification of genetic associations between acute myocardial infarction and non-small cell lung cancer

**DOI:** 10.3389/fmolb.2024.1502509

**Published:** 2024-12-06

**Authors:** Hao Zheng, Jie Wang, Yijia Zheng, Xiaofan Hong, Luxi Wang

**Affiliations:** ^1^ First School of Clinical Medicine, Wenzhou Medical University, Wenzhou, China; ^2^ Wenzhou Medical University, Wenzhou, China; ^3^ Department of Psychiatry, Wenzhou Seventh People’s Hospital, Wenzhou, China; ^4^ Department of Neurology, The First Affiliated Hospital of Wenzhou Medical University, Wenzhou, China

**Keywords:** non-small cell lung cancer (NSCLC), acute myocardial infarction (AMI), differentially expressed genes (DEGs), hub genes, bioinformatics analysis

## Abstract

**Introduction:**

A growing body of evidence suggests a potential connection between myocardial infarction (MI) and lung cancer (LC). However, the underlying pathogenesis and molecular mechanisms remain unclear. This research aims to identify common genes and pathways between MI and LC through bioinformatics analysis.

**Methods:**

Two public datasets (GSE166780 and GSE8569) were analyzed to identify differentially expressed genes (DEGs). Common DEGs were enriched using Gene Ontology (GO) and the Kyoto Encyclopedia of Genes and Genomes (KEGG). Hub genes were identified and their diagnostic performance was evaluated. Gene co-expression networks, as well as regulatory networks involving miRNA-hub genes and TF-hub genes, were also constructed. Finally, candidate drugs were predicted.

**Results:**

Among the datasets, 34 common trend DEGs were identified. Enrichment analysis linked these DEGs to key biological processes, cellular components, and molecular functions. Eight hub genes (*CEBPA*, *TGFBR2*, *EZH2*, *JUNB*, *JUN*, *FOS*, *PLAU*, *COL1A1*) were identified, demonstrating promising diagnostic accuracy. Key transcription factors associated with these hub genes include *SP1*, *ESR1*, *CREB1*, *ETS1*, *NFKB1*, and *RELA*, while key miRNAs include hsa-mir-101-3p, hsa-mir-124-3p, hsa-mir-29c-3p, hsa-mir-93-5p, and hsa-mir-155-5p. Additionally, potential therapeutic drugs were identified, with zoledronic acid anhydrous showing potential value in reducing the co-occurrence of the two diseases.

**Discussion:**

This study identified eight common signature genes shared between NSCLC and AMI. Validation datasets confirmed the diagnostic value of key hub genes *COL1A1* and *PLAU*. These findings suggest that shared hub genes may serve as novel therapeutic targets for patients with both diseases. Ten candidate drugs were predicted, with zoledronic acid showing potential for targeting dual hub genes, offering a promising therapeutic approach for the comorbidity of lung cancer and myocardial infarction.

## 1 Introduction

Lung cancer (LC), a leading cause of cancer-related death globally (Shtivelman et al., 2014), encompasses small cell lung cancer (SCLC) as well as non-small cell lung cancer (NSCLC), which includes lung adenocarcinoma (LUAD), lung squamous cell carcinoma (LSCC), and large cell carcinoma (Sahu et al., 2023). NSCLC accounts for approximately 85% of all LC diagnoses (Stolz et al., 2022). Though studies in epidemiology have clearly demonstrated that cigarette smoke (CS) exposure contributes to the risk of NSCLC (Hecht, 1999), and many studies have focused on identifying specific targets for NSCLC, such as *PIK3CA* (Yamamoto et al., 2008), *PDGFRA* (Ramos et al., 2009), *EPHA2* (Psilopatis et al., 2022), etc., the precise etiology and pathogenesis remain elusive.

Myocardial infarction (MI) is a form of cardiac injury caused by inadequate blood supply and oxygen deprivation (Murphy and Goldberg, 2022), usually associated with the ongoing development of arterial plaque over time. The main pathological characteristic is the impairment of endothelial function (Zhang et al., 2024). Acute myocardial infarction (AMI) is the main factor contributing to deaths among cardiovascular diseases, with increasing incidence and fatality rates (Huang et al., 2024). Studies have revealed associations between MI and processes such as pyroptosis, apoptosis, and PAN apoptosis (Chang et al., 2024).

There are certain associations between MI and LC. Firstly, these two diseases share common risk factors such as smoking (Ambrose and Barua, 2004) and personality type (Nagano et al., 2001). In terms of epidemiology, patients with lung malignancies show an increased incidence of cardiovascular (CV) events (Mitchell et al., 2023). Additionally, recent studies have shown that the heart, especially myocardial mesenchymal stromal cells, releases extracellular vesicles with tumor characteristics after MI, and their tumor-promoting effects have a greater impact on LC (Caller et al., 2024). This provides further evidence for the link between MI and LC. The side effects of NSCLC treatment also have a certain impact. Platinum is associated with the occurrence of AMI, and tyrosine kinase inhibitors have shown certain cardiotoxicity (Chang et al., 2023). It is reported that the incidence of vascular events in patients with NSCLC treated with immune checkpoint inhibitors cannot be ignored (Giustozzi et al., 2021). It is worth noting that NSCLC and AMI share some common pathogenesis. As for the inflammatory response, the occurrence and development of AMI is closely related to abnormal inflammatory cells (Sun et al., 2024) and persistent inflammation will increase the risk of lung cancer (Elsayed, 2024). In addition, oxidative stress (Di Carlo and Sorrentino, 2024; Guo et al., 2024), vascular remodeling and endothelial dysfunction (Li et al., 2023; Meng et al., 2021) are involved in the pathogenesis of both diseases. However, the specific pathogenesis and molecular mechanisms underlying the association between MI and LC have not yet been fully understood.

**FIGURE 1:**
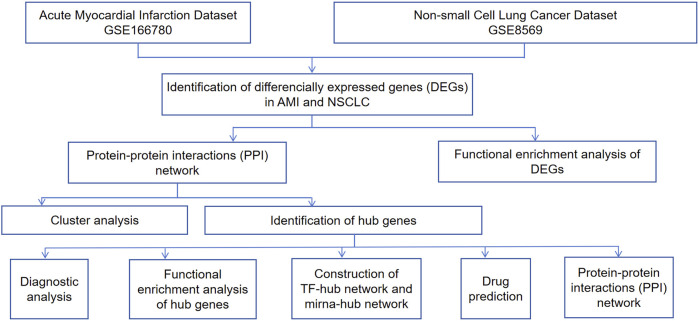
Flowchart of the study.

Microarray technology and network-based analysis provide valuable insights into gene expression profiles across various cancers. In this research, we performed a bioinformatics analysis to detect shared molecular mechanism-based biomarkers (*CEBPA*, *TGFBR2*, *EZH2*, *JUNB*, *JUN*, *FOS*, *PLAU*, *COL1A1*) between NSCLC and AMI, validating their correlation and identifying candidate drugs targeting hub genes. These hub genes may be involved in the onset and progression of AMI and NSCLC through inflammatory response, oxidative stress, apoptosis, and related signaling pathways. In NSCLC and AMI, *COL1A1* is involved through EMT and arterial dissection, while *PLAU* contributes through tumor invasiveness and macrophage function, each separately. Notably, *COL1A1* and *PLAU* are dual targets of zoledronic acid anhydrous, a drug proven in previous experiments to reduce cancer mortality and the incidence of cardiovascular events. Our study suggests that zoledronic acid anhydrous has promising potential in the combined treatment of AMI and NSCLC. This study offers insights into the formulation of dual-purpose preventative and therapeutic approaches. The research process is illustrated in Figure 1.

## 2 Materials and methods

### 2.1 Data collection

The Gene Expression Omnibus (GEO) is the largest and most comprehensive public gene expression data resource (https://www.ncbi.nlm.nih.gov/geo/). We used the keywords “lung cancer” and “myocardial infarction” to identify datasets related to NSCLC and MI. The selected datasets were based on the following criteria: they must cover both cases and controls, samples must be from human subjects, and they must provide original information for subsequent analysis. Finally, we chose the NSCLC-related dataset GSE8569 and the AMI-related dataset GSE166780 for subsequent research. Furthermore, we included the NSCLC-related dataset GSE75037 and the AMI-related dataset GSE34198 for validation. Detailed information on these datasets is provided in Table 1.

**TABLE 1:** Clinical characteristics.

	GSE166780			GSE8569			GSE75037^a^			GSE34198		
	N	AMI	P	LC	N	P	N	LC	P	N	AMI	P
Sample Count	8	8		69	6		83	83		48	45	
Age^b^ (years)	59.75 ± 8.71	63.25 ± 8.29	0.424	NA	NA	NA	68.11 ± 9.74	68.11 ± 9.74	1.000	65.69 ± 9.30	63.62 ± 9.24	0.286
Female (%)	38	25	1.000	14	NA	NA	71	71	1.000	33	31	0.819
Ancestry (%)			NA			NA			1.000			NA
Asian	NA	NA		NA	NA		30	30		NA	NA	
European	NA	NA		NA	NA		67	67		NA	NA	
American Indian or Alaska Native	NA	NA		NA	NA		1	1		NA	NA	
Source Name	Peripheral Blood Monocyte			Lung			Lung			Peripheral Whole Blood		

^a^ Tumor tissue and normal tissue are from the same patient.

^b^ Data are presented as mean ± standard deviation.

Abbreviations: N, normal; AMI, acute myocardial infarction; P, *p*-value; LC, lung cancer; NA, not available.

### 2.2 Identification of differentially expressed genes (DEGs)

The online analysis tool GEO2R was utilized to identify DEGs, using screening criteria of |log2(FC)| > 1 and *p* < 0.05. DEGs specific to NSCLC and AMI were obtained by comparing gene expression profiles between NSCLC cancer cells and normal cells, as well as between peripheral blood samples from AMI patients and normal peripheral blood samples. The Venn diagram tool of the Xiantao Academic Platform (https://www.xiantaozi.com) was used to find the common DEGs of the two diseases. The results were visualized using volcano plots, box plots, and Venn diagrams.

### 2.3 Enrichment analysis of DEGs

First, the common DEGs were converted to gene IDs, followed by gene enrichment analysis. Gene Ontology (GO) analysis, encompassing biological processes (BP), molecular function (MF), and cellular component (CC), is a widely used gene annotation bioinformatics tool. Kyoto Encyclopedia of Genes and Genomes (KEGG) provides comprehensive information on genomes, biological pathways, diseases, and chemicals. Using the clusterProfiler package in RStudio, we conducted GO functional enrichment and KEGG pathway analyses on the common DEGs. The ggplot2 package was used to visualize the data. The filtering condition was set at *p* < 0.05.

### 2.4 Protein-protein interaction (PPI) network construction

We used the STRING database (https://cn.string-db.org/) to construct a PPI network for the co-expressed DEGs in NSCLC and AMI. The minimum interaction score was set to 0.400 and the FDR stringency for filtering was set to 0.500 to identify interactions among protein-coding genes. Then, we processed the data using Cytoscape 3.10.2, clustered the gene network using the “MCODE” plug-in, identified key subnetwork modules, and performed cluster analysis. Hub genes were determined using the cytohubba method (Degree, EPC, MCC, and MNC). We constructed a hub gene expression network using the GeneMANIA database (http://www.genemania.org/).

### 2.5 Diagnostic significance of hub genes

We further investigated the importance of key genes as potential biomarkers. The receiver operating characteristic (ROC) curve was employed to assess the sensitivity and specificity of our selected target genes. The area under the ROC curve (AUC) was used to evaluate the results.

### 2.6 Construction of miRNA-hub gene network and TF-hub gene network

The online database NetworkAnalyst 3.0 (https://www.networkanalyst.ca/) was used to construct the miRNA-hub and TF-hub networks of hub genes to describe the associations between hub genes, miRNA, and TF. The selected TF-gene interaction database was ENCODE, using criteria of peak intensity signal <500 and the predicted regulatory potential score <1. The miRNA-gene interaction data were collected from miRTarBase.

### 2.7 Drug prediction based on hub genes

We searched DGIdb (https://dgidb.genome.wustl.edu), a database for exploring known and potential drug-gene interactions, to identify candidate drugs associated with the hub genes.

### 2.8 Statistical analyses

Statistical analyses were performed using SPSS version 27. Clinical characteristics were compared using Student’s t-test, Pearson Chi-Square test or Fisher’s exact test, as appropriate. A two-sided *p*-value of less than 0.05 was considered statistically significant.

## 3 Results

### 3.1 Identification of DEGs

**FIGURE 2:**
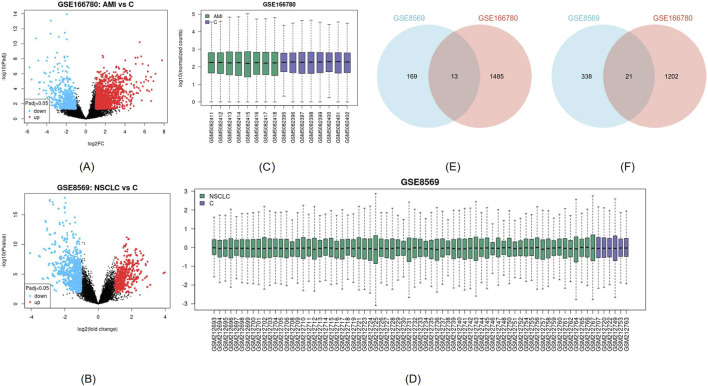
**(A, B)** Visualization of DEGs of GSE166780 and GSE8569 using volcano plots, blue represents downregulation, red represents upregulation (|log2(FC)|>1, *p* < 0.05) **(C, D)** Visualization of GSE166780 and GSE8569 using box plots **(E)** Co-expressed upregulated differentially expressed genes (DEGs) of GSE166780 and GSE8569 (|log2(FC)|>1, *p* < 0.05) **(F)** Co-expressed downregulated DEGs of GSE166780 and GSE8569 (|log2(FC)|>1, *p* < 0.05).

The AMI-related dataset GSE166780 contains 8 normal and 8 AMI samples, while the NSCLC-related dataset GSE8569 includes 69 NSCLC and 6 normal samples. We compared the gene expression of AMI and normal samples in GSE166780 and identified 3060 AMI DEGs, including 1,498 upregulated genes and 1,223 downregulated genes (Figures 2A, C). Similarly, we compared the gene expression of normal and NSCLC samples in GSE8569 and identified 678 NSCLC DEGs, including 182 upregulated genes and 359 downregulated genes (Figures 2B, D). Through the Venn diagram, 13 co-upregulated DEGs and 21 co-downregulated DEGs were filtered out (Figures 2E, F). The co-expressed genes and their corresponding *p*-values are listed in Table 2.

**TABLE 2:** Differential expression genes.

DEG	AMI		NSCLC		DEG	AMI		NSCLC	
	P	L2FC	P	L2FC		P	L2FC	P	L2FC
*PRC1*	0.0105	1.76	0.0003	1.69	*MMP14*	0.0015	3.08	0.0389	1.09
*PSME4*	0.0030	1.57	<0.0001	1.02	*HYAL2*	0.0256	−1.84	<0.0001	−1.85
*PTTG1*	0.0124	1.86	0.0007	1.51	*MAP3K11*	0.0241	−1.35	<0.0001	−2.01
*MCM6*	0.0024	1.69	0.0001	1.31	*JUNB*	0.0408	−1.57	<0.0001	−1.99
*EZH2*	<0.0001	2.99	0.0008	1.31	*DAPK2*	0.036	−1.37	0.0001	−1.08
*PPIF*	0.0020	1.81	0.0056	1.07	*FLI1*	0.0314	−1.17	<0.0001	−1.53
*COL1A1*	0.0137	2.59	0.0005	2.87	*FOS*	0.037	−1.20	<0.0001	−2.70
*HMGA1*	0.047	1.36	0.0009	1.59	*GNAI2*	0.0296	−1.34	<0.0001	−1.10
*TNFRSF21*	0.0103	1.76	0.0045	1.23	*TGFBR2*	0.0078	−1.01	<0.0001	−1.22
*CFB*	<0.0001	6.90	0.0074	1.06	*PINK1*	0.0004	−1.59	<0.0001	−1.03
*PLAU*	<0.0001	6.15	0.0147	1.36	*RHOB*	0.0019	−2.17	0.0001	−1.07
*MAFG*	0.0002	2.74	0.0060	−1.24	*CSF3R*	0.0246	−1.13	0.0002	−1.17
*PAQR8*	0.0107	−2.24	0.0002	−1.21	*CITED2*	0.026	−1.69	0.0030	−1.32
*FKBP8*	0.0168	−1.30	0.0005	−1.07	*GIMAP6*	0.0363	−1.51	0.0041	−1.31
*JUN*	0.0382	−1.20	0.0011	−1.18	*ZYX*	0.0412	−1.21	0.0063	−1.05
*IMPDH1*	0.0335	−1.24	0.0011	−1.30	*CEBPA*	<0.0001	−3.40	0.0165	−1.32
*TLN1*	0.0083	−1.24	0.0016	−1.20	*HLA-G*	0.0142	−1.43	0.0178	−1.47

Abbreviations: P, *p*-value; L2FC, log2(FC).

### 3.2 Functional annotation analysis of DEGs

**FIGURE 3:**
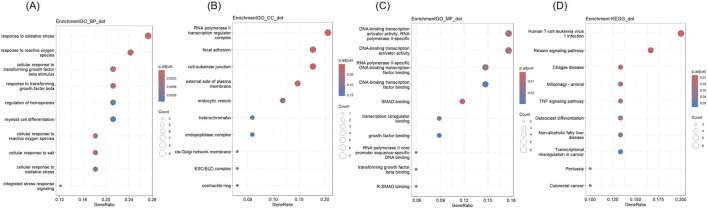
Gene Ontology (GO) and Kyoto Encyclopedia of Genes and Genomes (KEGG) analysis of DEGs **(A)** Biological Processes **(B)** Cellular Component **(C)** Molecular Function **(D)** KEGG.

GO and KEGG analysis were conducted on 34 co-expressed DEGs in NSCLC and AMI, and the outcomes are depicted in Figure 3. Concerning the BP, DEGs were significantly concentrated in “response to oxidative stress” and “response to reactive oxygen species.” In terms of CC, DEGs were enriched in “RNA polymerase II transcription regulator complex,” “focal adhesion,” and “cell-substrate junction.” For MF, DEGs are enriched in “DNA-binding transcription activator activity, RNA polymerase II-specific,” and “DNA-binding transcription activator activity.” KEGG analysis revealed that DEGs were enriched in “Human T-cell leukemia virus 1 infection” and “Relaxin signaling pathway.”

### 3.3 PPI network analysis, cluster analysis, and identification of hub genes

**FIGURE 4:**
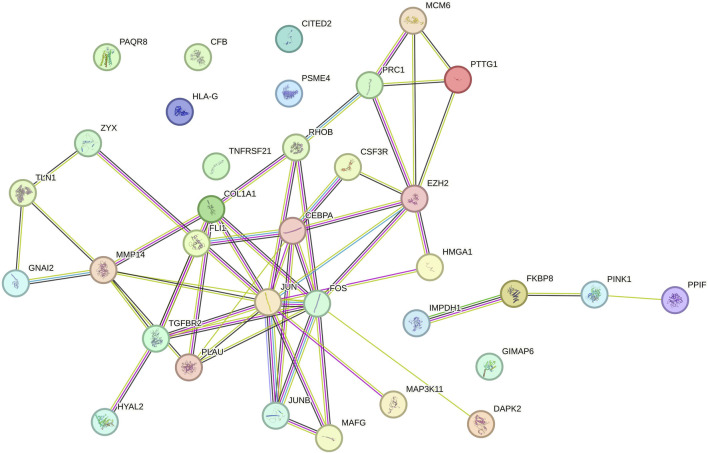
Protein-protein interaction (PPI) network of common DEGs.

**FIGURE 5:**
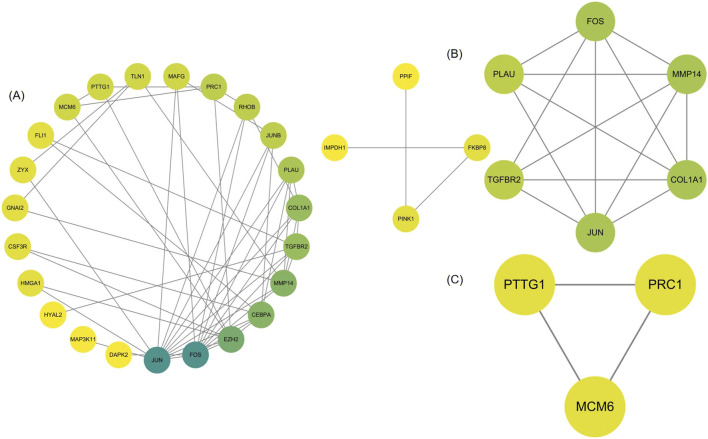
**(A)** PPI network of common DEGs visualized using Cytoscape **(B, C)** MCODE plugin shows that cluster 1 contains 6 nodes and 14 edges with a score of 5.6. Cluster 2 contains 3 nodes and 3 edges with a score of 3.

**FIGURE 6:**
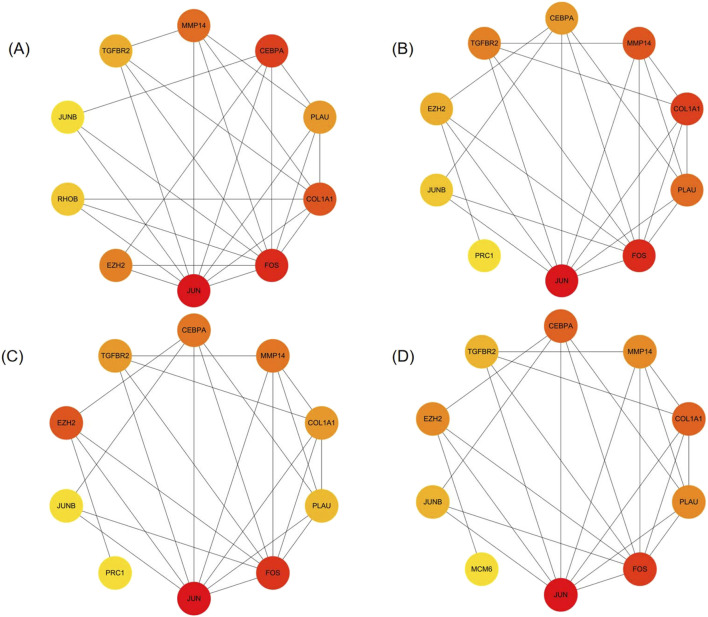
Identification of hub genes **(A–D)** The top 10 genes in the PPI network were sorted by Degree, MCC, EPC, and MNC respectively. The 8 hub genes identified are *CEBPA, TGFBR2, EZH2, JUNB, JUN, FOS, PLAU, COL1A1*.

We analyzed 34 shared DEGs using the STRING online database (Figure 4) and imported the data into Cytoscape for visualization. After removing isolated points, the PPI of the shared DEGs is illustrated in Figure 5A, with 27 nodes and 53 edges. Additionally, we employed the MCODE plug-in to identify crucial gene cluster modules and discovered 2 clusters. Cluster 1 contains 6 nodes and 14 edges with a score of 5.6. Cluster 2 includes 3 nodes and 3 edges with a score of 3 (Figures 5B, C). To identify hub genes, we used the CytoHubba plug-in. By taking the intersection of the four algorithms, we identified 8 hub proteins: *CEBPA*, *TGFBR2*, *EZH2*, *JUNB*, *JUN*, *FOS*, *PLAU* and *COL1A1* (Figure 6). In the NSCLC and AMI datasets, *EZH2*, *COL1A1* and *PLAU* were upregulated, and *CEBPA*, *TGFBR2*, *JUN*, *JUNB* and *FOS* were downregulated.

### 3.4 Enrichment analysis of hub genes

**FIGURE 7:**
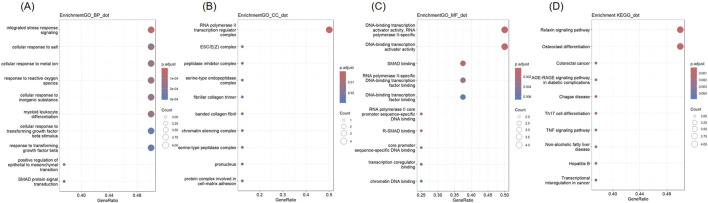
GO and KEGG analysis of Hub genes **(A)** Biological Processes **(B)** Cellular Component **(C)** Molecular Function **(D)** KEGG.

GO and KEGG analyses were conducted (Figure 7). The hub genes were mainly related to “integrated stress response signaling,” “cellular response to salt,” “cellular response to metal iron,” “response to reactive oxygen species” “cellular response to inorganic substance,” “myeloid leukocyte differentiation,” “cellular response to transforming growth factor beta” in BP terms. In CC terms, they were predominantly associated with the “RNA polymerase II transcription regulator complex.” In MF terms, they were mainly related to “DNA-binding transcription activator activity, RNA polymerase II-specific,” “DNA-binding transcription activator activity,” “SMAD binding,” “RNA polymerase II-specific DNA-binding transcription factor binding,” and “DNA-binding transcription factor binding.” In KEGG analysis, they were enriched in “Relaxin signaling pathway,” and “Osteoclast differentiation.”

### 3.5 PPI analysis of hub genes

**FIGURE 8:**
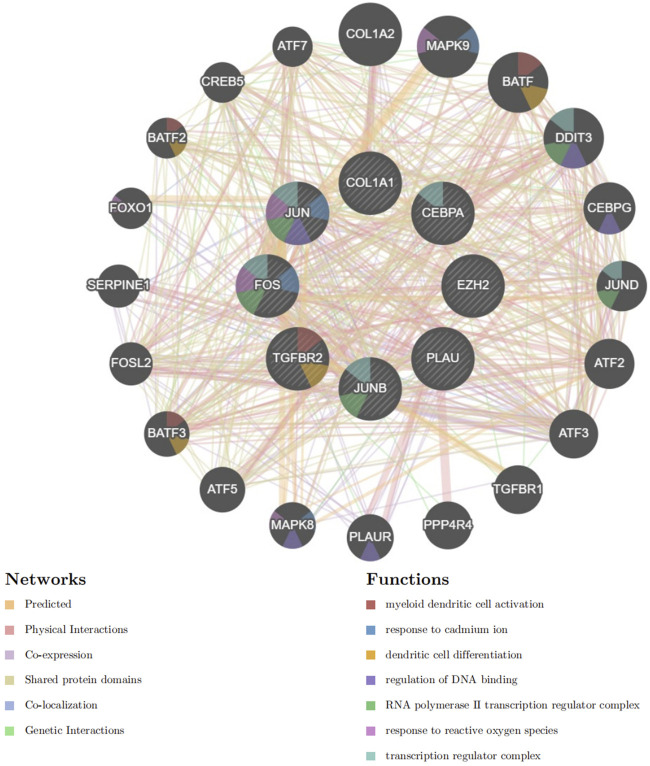
PPI analysis of hub genes using GeneMANIA.

Based on GeneMANIA, PPI analysis was performed on 8 hub genes and 20 cross genes (Figure 8). Among them, predicted accounted for 32.57%, physical Interactions for 28.18%, co-expression for 28.02%, shared protein domains for 10.32%, co-localization for 0.73% and genetic interactionsfor 0.18%. The primary biological roles of this network pertain to the myeloid dendritic cell activation, response to cadmium ion, dendritic cell differentiation, regulation of DNA binding, RNA polymerase II transcription regulator complex, response to reactive oxygen species and transcription regulator complex.

### 3.6 Diagnostic significance of hub genes

**FIGURE 9:**
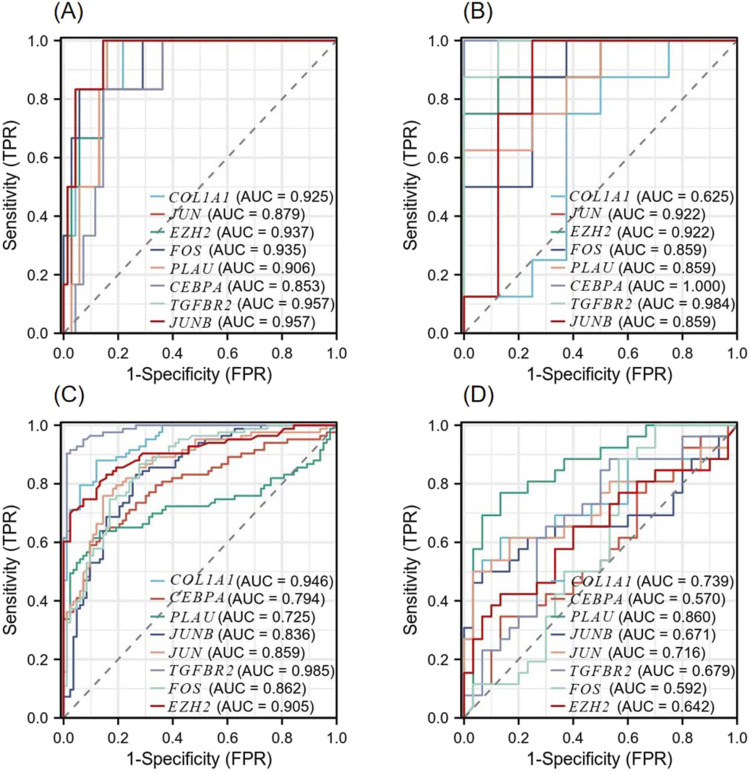
Diagnostic Receiver operating characteristic (ROC) curves of 8 co-expressed hub genes **(A)** ROC curve of the hub gene in the GSE8569 dataset. **(B)** ROC curve of the hub gene in the GSE166780 dataset. **(C)** ROC curve of the hub gene in the GSE75037 dataset. **(D)** ROC curve of the hub gene in the GSE34198 dataset.

According to the ROC curve developed by the eight candidate hub genes, we evaluated the specificity and sensitivity of each gene for diagnosis (Figure 9). We calculated the AUC of each item, among which the AUCs of *COL1A1*, *JUN*, *EZH2*, *FOS*, *PLAU*, *CEBPA*, *TGFBR2*, and *JUNB* in the NSCLC-related dataset were 0.925, 0.879, 0.937, 0.935, 0.906, 0.853, 0.957, and 0.957, respectively. The AUCs of *COL1A1*, *JUN*, *EZH2*, *FOS*, *PLAU*, *CEBPA*, *TGFBR2*, and *JUNB* in the AMI-related dataset were 0.625, 0.922, 0.922, 0.859, 0.859, 1.000, 0.984, and 0.859, respectively. These genes have high diagnostic value for both NSCLC and AMI. Among them, the AUC of *EZH2* and *TGFBR2* were greater than 0.9 in both diseases, showing high diagnostic efficacy. In the validation dataset of NSCLC, the AUCs of *COL1A1*, *CEBPA*, *PLAU*, *JUNB*, *JUN*, *TGFBR2*, *FOS*, and *EZH2* were 0.946, 0.794, 0.725, 0.836, 0.859, 0.985, 0.862, and 0.905, respectively. In the validation dataset of AMI, the AUCs of *COL1A1*, *CEBPA*, *PLAU*, *JUNB*, *JUN*, *TGFBR2*, *FOS*, and *EZH2* were 0.739, 0.570, 0.860, 0.671, 0.716, 0.679, 0.592, and 0.642, respectively. In both validation datasets, *COL1A1*, *PLAU*, *JUNB*, *JUN*, *TGFBR2*, and *EZH2* maintained high diagnostic efficacy.

### 3.7 Construction of TF-hub gene network and miRNA-hub gene network

**FIGURE 10:**
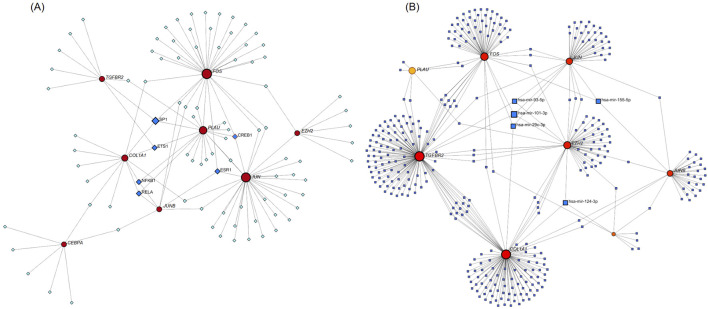
**(A)** TF-Hub genes network, circles are hub genes, and squares are TFs. **(B)** miRNA-Hub genes network, circles are hub genes, and squares are miRNAs.

We used NetworkAnalyst 3.0 software to construct the TF-Hub genes and miRNA-Hub genes regulatory networks. The TF-Hub network consists of 100 nodes and 121 edges, and the miRNA-Hub network has 358 nodes and 407 edges. We found that in the TF-Hub network, *SP1* interacts with *TGFBR2*, *FOS*, *PLAU*, and *COL1A1*. *ESR1* regulates *FOS*, *JUN*, and *JUNB*. *CREB1* regulates *PLAU*, *JUN*, and *FOS*. *ETS1* interacts with *COL1A1*, *TGFBR2*, and *PLAU*. Notably, *NFKB1* and *RELA* simultaneously regulate *COL1A1*, *JUNB* and *PLAU* (Figure 10A). In the miRNA-Hub network, hsa-mir-101-3p interacts with 4 genes, including *JUN*, *EZH2*, *TGFBR2* and *FOS*. Hsa-mir-124-3p targets *EZH2*, *COL1A1*, and *CEBPA*. Hsa-mir-29c-3p targets *JUN*, *FOS*, and *COL1A1*. Hsa-mir-93-5p targets *JUN*, *TGFBR2*, and *EZH2*. Hsa-mir-155-5p targets *JUN*, *JUNB*, and *FOS* (Figure 10B).

### 3.8 Identification of drug candidates

**FIGURE 11:**
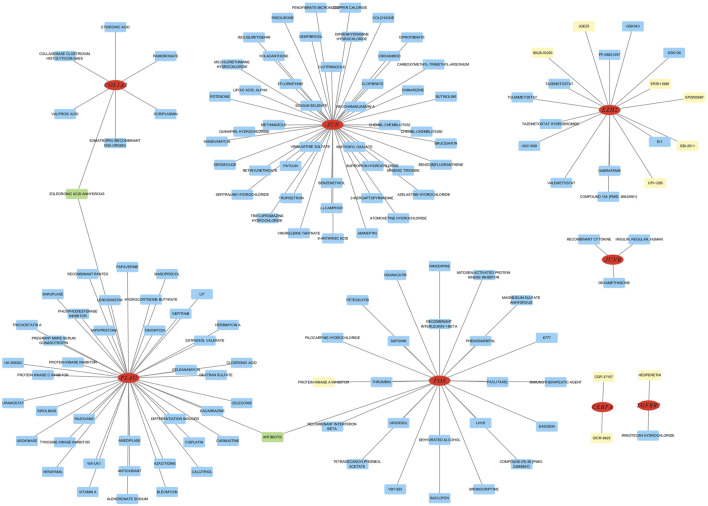
Analysis of hub genes on DGibd, a sum of 145 drugs were discovered. Red indicates hub genes, yellow indicates the top ten drugs, green indicates drugs connected with two genes, and blue indicates other potential drugs.

To investigate gene-drug interactions, 8 validated hub genes were submitted to the DGIdb database, and the results were visualized in Cytoscape (Figure 11). A total of 145 drugs were identified, and the top ten were CGP-37157, OICR-9429, EPZ005687, EPZ011989, HESPERETIN, PROTEIN KINASE A INHIBITOR, JQEZ5, SKLB-03220, EBI-2511, CPI-1205 (Table 3). It is worth noting that zoledronic acid anhydrous and antibiotic are connected with two hub genes.

**TABLE 3:** The top ten drugs selected by DGIdb according to the score ranking.

Drug	Interaction type and directionality	Interaction score	Target gene
CGP-37157	inhibitor (INHIBITORY)	26.25	*CEBPA*
OICR-9429	n/a	13.12	*CEBPA*
EPZ005687	inhibitor (INHIBITORY)	11.66	*EZH2*
EPZ011989	inhibitor (INHIBITORY)	5.83	*EZH2*
HESPERETIN	n/a	5.83	*TGFBR2*
PROTEIN KINASE A INHIBITOR	n/a	4.2	*FOS*
JQEZ5	inhibitor (INHIBITORY)	2.91	*EZH2*
SKLB-03220	inhibitor (INHIBITORY)	2.91	*EZH2*
EBI-2511	inhibitor (INHIBITORY)	2.91	*EZH2*
CPI-1205	inhibitor (INHIBITORY)	2.91	*EZH2*

## 4 Discussion

Previous studies have demonstrated an inverse relationship between cardiovascular disease and cancer. The mechanism behind this relationship is not fully understood. Still, it may be associated with circulating microRNAs, extracellular vesicles, cardiac-derived mediators, along with pathways associated with inflammatory responses, clonal hematopoiesis, and oxygen deprivation (Aboumsallem et al., 2020). Several studies have indicated an increased risk of cancer in patients with MI (Hasin et al., 2016; Rinde et al., 2017). Research into the common mechanisms between MI and cancer has gained attention recently (Yuan et al., 2023; Meijers et al., 2018). However, limited studies have explored the shared genetic underpinnings between NSCLC and AMI at the genetic level. Our study has examined the molecular biological roles and common pathways of NSCLC and AMI, offering insights for the development of dual-purpose prevention and therapy strategies.

In this study, we utilized bioinformatics tools to identify 34 common DEGs between an NSCLC dataset and an AMI dataset. GO analysis of these DEGs revealed their association with “response to reactive oxygen species,” “response to oxidative stress,” “RNA polymerase II transcription regulator complex,” “focal adhesion,” “cell-substrate junction,” “RNA polymerase II-specific DNA binding transcription factor binding,” “DNA-binding transcription activator activity, RNA polymerase II-specific.” KEGG analysis revealed that these DEGs were linked to pathways involving “Human T-cell leukemia virus 1 infection,” and “Relaxin signaling pathway.”

We developed a PPI network of DEGs and detected 8 hub genes, namely, *CEBPA*, *TGFBR2*, *EZH2*, *JUNB*, *JUN*, *FOS*, *PLAU* and *COL1A1*. In both datasets, *EZH2*, *COL1A1* and *PLAU* were upregulated, while *CEBPA*, *TGFBR2*, *JUN*, *JUNB* and *FOS* were downregulated.

*COL1A1* encodes the pro-α1 chain of type I collagen, a fibrillogenic collagen found abundantly in bone, cornea, dermis, and tendon. It has been recognized as a predictive biomarker for LUAD, and its increased expression has been observed in LC tissue samples, consistent with our findings (Dong et al., 2023). EMT and fibroblast-myofibroblast-myofibroblast transition (FMT) are involved in the initiation and progression of cancer (Ribatti et al., 2020), and studies have demonstrated that fibroblasts with high levels of *COL1A1* are related to EMT (Wang et al., 2022). This means that highly expressed *COL1A1* may participate in the onset and progression of LC by participating in the EMT process. Spontaneous coronary artery dissection (SCAD) constitutes a non-atherosclerotic etiology for AMI (Saw et al., 2017). Studies have linked *COL1A1* to the formation of arterial dissection (Zekavat et al., 2022), which may explain the association between AMI and *COL1A1*. Some studies have also pointed out that type I collagen’s effective deposition is crucial for the healing process after MI (Nong et al., 2011). More research is still needed to elucidate the underlying mechanism.

*CEBPA* contributes to the development, expansion, metabolic functions, and immune responses (Newman and Keating, 2003). It has been reported to be downregulated in various solid tumors, including liver, breast cancer, and LC (Lourenco and Coffer, 2017), similar to our findings. *CEBPα* is reported to act as a key factor in preserving the stability of the epithelial cell layer by suppressing the transcription of vital mesenchymal markers, which in turn stops the initiation of tumors driven by the EMT process (Lourenco et al., 2020). Studies have shown that therapeutic upregulation of *CEBPA* leads to inactivation of immunosuppressive myeloid cells and has effective anti-tumor responses in different tumor models and cancer patients (Hashimoto et al., 2021). Furthermore, some scholars have demonstrated that JAM-A activates *CEBPα* and induces the transcription of the claudin-5 gene, which plays a key role in maintaining the vascular barrier (Kakogiannos et al., 2020). However, there are a few reports that deviate from this trend (Zhu et al., 2024), and it is reported that *CEBPA* can promote *LOXL2* and *LOXL3* transcription and stabilize *BCL-2*, thereby enhancing the proliferation and metastasis of LC cells *in vitro* (Fan et al., 2024). In short, the function of *CEBPA* in the genesis and progression of tumors still needs further confirmation. In cardiac research, studies have revealed that the C/EBP family’s transcription factors (TFs) are activated in the epicardium upon receiving developmental prompts and injury indicators. They collaborate with *HOX*, *MEIS*, and Grainyhead TFs to clarify the genomic program directing embryonic gene transcription in the epicardium (Huang et al., 2012). However, the relationship between *CEBPA* and AMI has not been fully elucidated.

*FOS* is a member of the *FOS* gene family. The protein encoded by *FOS* can form the TF complex AP-1 with the *Jun* family proteins through the leucine zipper and is vital in signal transduction, cell proliferation, and differentiation (Li et al., 2024). A study has shown that the levels of *c-fos* and *c-jun* in tumor tissues of NSCLC cases are lower than those in adjacent normal tissues (Levin et al., 1994). It is reported that *FOS* is downregulated in cases of heart failure (HF) after MI as well (Hu et al., 2023). Our results similarly found that *FOS* was downregulated in both diseases. However, some reports show the opposite. As a target for miR-101A, overexpression of *FOS* can lead to aggravated myocardial fibrosis after MI (Pan et al., 2012), and inhibition of Fos/AP-1 can reduce inflammatory response and cardiac dysfunction (Zhuang et al., 2022). At the same time, miR-29b-3p mitigates cardiac fibrosis following infarction by directly targeting *FOS* (Xue et al., 2020). Studies have also found that focusing on *c-fos* may be a supplementary treatment approach to hinder and diminish metastasis in LUAD patients carrying SNP BRMS1v2 A273V/A273V (Liu et al., 2022). Therefore, the association between the *FOS* gene and the two diseases requires further study.

*JUN* is a potential oncogene of avian sarcoma virus 17. Dysregulation of the mitogen-activated protein kinase (MAPK) signaling pathway is critical in the progression of LC and several other cancer types (Braicu et al., 2019). The c-Jun N-terminal kinase (JNK) pathway, one of the MAPK pathways, is involved in various cellular functions in tumor development, including proliferation, differentiation, survival, and apoptosis (Johnson and Lapadat, 2002). Mesothelin (MSLN) promotes the level and activation of MET through the JNK signaling pathway, enabling cancer cells to disrupt tight junctions and the integrity of the blood-brain barrier (BBB), thus penetrating the barrier (Xia et al., 2024). Studies have also demonstrated that c-Jun is essential for coordinating the developmental processes of cardiac cells during their early stages (Su et al., 2023). Activation of JNK has been linked to myocardial injury, left ventricular remodeling (LVR), and HF after MI (Plotnikov et al., 2023). In our study, *JUN* was downregulated in both AMI and NSCLC, which may be related to these mechanisms and thus supporting these findings to a certain extent.

*EZH2* is an important catalytic protein and is part of the polycomb repressive complex 2 (PRC2) family. In our study, *EZH2* was upregulated in both diseases. Previous studies have demonstrated that *EZH2* is elevated in ischemic hearts (Zhao et al., 2021). In the context of MI, inhibition of *EZH2*-induced cardiac recruitment and enhanced activity of non-classical monocytes accelerates the resolution of inflammation and reduces infarct scar expansion, thereby contributing to decreased cardiac remodeling and dysfunction after MI (Rondeaux et al., 2023). Exosomal miR-25-3p from mesenchymal stem cells reduces MI by inhibiting *EZH2* (Peng et al., 2020). *EZH2* is a target of *SETD1A*, which maintains cancer stem cell properties by triggering Wnt/β-catenin pathway activity (Wang R. et al., 2021). It has been proposed that inhibition of *EZH2* combined with inhibition of *PI3K* is a possible combination therapy against LC with *PIK3CA* alteration or overexpression (Chen et al., 2022). Previous studies have shown that BMSC-exo-miR-30b-5 can regulate the development of NSCLC by targeting *EZH2* (Wu et al., 2023).

*PLAU* is a urokinase plasminogen activator known for its role in cancer invasiveness, positioning it as a central figure in cancer metastasis and related invasion processes, including attachment, movement, and infiltration (Han et al., 2005; Sliva, 2008). Our results showed that *PLAU* was upregulated in both diseases. *PLAU* is closely associated with mutations in the tumor suppressor gene *TP53*, which prevents the occurrence of anoikis (Barta et al., 2020). *PLAU* has been associated with a wide range of biological and pathological mechanisms, covering chemotaxis, adhesion processes, migration and growth (Chavakis et al., 2002). Overexpression of *PLAU* positively regulates the growth and colony formation of NSCLC cells (Zheng et al., 2024). Elevated *PLAU* levels in macrophages accelerate atherosclerosis and blockage of the coronary arteries (Cozen et al., 2004).

*TGFBR2,* a central component of the TGF-β pathway, is often deleted during carcinogenesis in numerous cancer types, including NSCLC (Wang et al., 2007), and functions as an effective tumor suppressor in NSCLC (Lo Sardo et al., 2021). We found that *TGFBR2* was downregulated in NSCLC and AMI. Masaki Ikeuchi et al. revealed that activation of TGF-β provides protective benefits against early ischemic heart damage. However, if its expression persists, the beneficial effects may be compromised, leading to LVR and failure after MI (Ikeuchi et al., 2004). Conversely, a study has demonstrated the potential benefits of targeting *TGFBR2* in alleviating MI-like symptoms *in vivo* and *in vitro* (Wang X. et al., 2021).

*JUNB* belongs to the family of activator protein-1 (AP-1) TFs and binds to specific sequences in the cis-regulatory domains of target genes, regulating multiple biological mechanisms encompassing cell division, cell growth, and programmed cell death. It has been reported that *JUNB* exhibited a substantial decrease in its mRNA and protein levels in cardiac tissues of HF mice (Yan et al., 2018). However, another study demonstrated that during early myocardial ischemia (EMI), *JUNB* increases in the nuclei of cardiomyocytes in both *in vivo* models and in human myocardium (Aljakna et al., 2018). The andrographolide can inhibit tumor growth and invasion of NSCLC by upregulating *HLJ1*, a novel tumor suppressor, through activation of *JUNB* (Lai et al., 2013). In this study, *JUNB* was downregulated in both diseases. In conclusion, research on the relationship between JUNB and these two diseases is limited, and more follow-up studies are needed.

GeneMANIA-based PPI analysis indicated that the primary biological functions of the hub genes and their interconnected genes are related to the myeloid dendritic cell activation, response to cadmium ion, dendritic cell differentiation, regulation of DNA binding, RNA polymerase II transcription regulator complex, response to reactive oxygen species and transcription regulator complex. Reactive oxygen species (ROS)-induced cardiomyocyte injury is critical for the pathogenesis of various heart diseases and involves multiple genes, TFs, and oxidation-sensitive signaling pathways (Li et al., 2013). MI and LC are both associated with oxidative stress. The cross-linking of cysteine residues 1,078 and 2,991 is essential for the redox control of the cardiac ryanodine receptor (RyR) (Nikolaienko et al., 2023). NOP56 and mTOR cooperate to maintain homeostasis in response to oxidative stress and significantly enhance cell death in KRAS mutant tumor cells (Yang et al., 2022).

ROC curve analysis was employed to assess the diagnostic efficacy of key genes, revealing their high diagnostic value for both NSCLC and AMI. Among these, *EZH2* and *TGFBR2* exhibited AUCs greater than 0.9 in both diseases. These findings underscore the significant roles of *COL1A1*, *JUN*, *EZH2*, *FOS*, *PLAU*, *CEBPA*, *TGFBR2*, and *JUNB* in the development of NSCLC and AMI. Subsequently, we used validation datasets to verify the diagnostic efficacy of hub genes and *COL1A1*, *PLAU*, *JUNB*, *JUN*, *TGFBR2* as well as *EZH2* maintaining high diagnostic efficacy. This further screened out genes with greater diagnostic value.

NetworkAnalyst was used to construct related miRNA-hub networks and TF-hub networks. The five most active miRNAs interacting with hub genes are hsa-mir-101-3p, hsa-mir-124-3p, hsa-mir-29c-3p, hsa-mir-93-5p, and hsa-mir-155-5p. The 6 TFs that predominantly interact with hub genes are *SP1*, *ESR1*, *CREB1*, *ETS1*, *NFKB1*, and *RELA*. These miRNAs and TFs could be correlated with the initiation and progression of NSCLC and AMI.

Previous studies have linked many of these miRNAs to the development of LC and AMI. For instance, miR-101-3p downregulation in cancer-associated fibroblasts (CAFs) increases vascular endothelial growth factor A (VEGFA) secretion, promoting LC metastasis via the Akt/eNOS pathway (Guo et al., 2021). Conversely, overexpression of miR-101a-3p can enhance cardiac function after MI (Li et al., 2019). In NSCLC, miR-124-3p markedly suppresses metastasis via exosomal transport and intracellular PI3K/AKT signaling (Zhu et al., 2023). Inhibition of miR-124-3p may activate the FGF21/CREB/PGC1α pathway, reducing cardiomyocyte apoptosis and improving oxidative stress and inflammatory responses (Wei et al., 2021). miR-29c-3p suppresses the activity of Mitochondrial fission regulator 1 (MTFR1), thereby inhibiting the progression of lung adenocarcinoma via the AMPK/mTOR signaling pathway (Li et al., 2021). miR-93-5p is often overexpressed in NSCLC and acts as an oncogene by inhibiting the tumor suppressor activities of PTEN and RB1 (Yang et al., 2018). The suppression of miR-155-5p rejuvenates senescent mesenchymal stem cells, thereby augmenting their cardioprotective effects post-myocardial infarction (Hong et al., 2020). Also, it can mediate the inhibition of radiotherapy in NSCLC (Zhu et al., 2019). In conclusion, these miRNAs are closely associated with both NSCLC and AMI.

There are also studies proving that predicted TFs are related to NSCLC and AMI. For example, HIF1α-SP1 interaction can promote the development of NSCLC (Wu et al., 2022). KN-93 enhances fatty acid oxidation in MI via the HDAC4-SP1 axis (Zhao et al., 2024). ESR1 expression serves as an independent prognostic indicator in metastatic NSCLC (Atmaca et al., 2014). ESR1 encodes the nuclear receptor ERα, which leads to a reduction in infarct size, inflammatory response, and oxidative stress in animal models (Puzianowska-Kuznicka, 2012). Inactivation of CREB1 may confer cisplatin resistance in metastatic NSCLC (Kim et al., 2019). *ETS1* mediates the initiation of gene expression patterns that result from the RAS/MAPK signaling pathway (Plotnik and Hollenhorst, 2017). The rs28362491 ins/del variation of the *NFKB1* gene is linked to a higher likelihood of MI and increased severity of coronary artery disease (Luo et al., 2020). *MUC3A* interacts with *RELA* to activate the NFκB pathway (Sun et al., 2021). In summary, these TFs could contribute to the onset and progression of the two diseases.

We used DGibd to predict dual-use drugs for AMI and NSCLC. A total of 145 candidate drugs were retrieved, of which the top ten scores were CGP-37157, OICR-9429, EPZ005687, EPZ011989, HESPERETIN, PROTEIN KINASE A INHIBITOR, JQEZ5, SKLB-03220, EBI-2511, and CPI-1205. Notably, zoledronic acid anhydrous and antibiotic target two hub genes. Zoledronic acid anhydrous targets *PLAU* and *COL1A1*, which are the two genes with the highest diagnostic value identified in our study. This offers a new perspective on the dual-use treatment of the two diseases. Some studies proved that zoledronic acid anhydrous may affect *COL1A1* expression. Specifically, treatment with bisphosphonates, including zoledronic acid anhydrous, inhibits expression levels of the *COL1A1* chains of type-I collagen in oral fibroblasts (Ravosa et al., 2011). A study indicates that women who received zoledronic acid treatment experienced fewer vascular events, lower cancer rates, and a tendency towards reduced mortality (Reid et al., 2020). This greatly confirms our view that zoledronic acid can be used in a dual-use treatment strategy for NSCLC and AMI. Zoledronic acid has also been reported to boost the effectiveness of immunotherapy in NSCLC (Zheng et al., 2022). This is a significant finding, as it suggests that zoledronic acid anhydrous could be a valuable adjunct to current immunotherapeutic approaches, potentially improving patient outcomes. Additionally, some antibiotics have been shown to affect *COL1A1* expression. The transgenes integrated at the *COL1A1* locus have demonstrated strong transgenerational inheritance of epigenetic alterations caused by fetal exposure to doxycycline (Wan et al., 2013), which is a tetracycline antibiotic. Scholars also found that the expression of *COL1A1* was significantly reduced in the rabbit model of benign tracheal stenosis treated with penicillin (Enyuan et al., 2018). However, it is reported that antibiotics can decrease the efficacy of immune checkpoint inhibitors in NSCLC treatment (Qiu et al., 2022). Therefore, the role of antibiotics in NSCLC remains to be determined. Evidence also suggests that among the top ten ranked drugs, some possess the potential for application in the treatment of both diseases. Some scholars have discovered that hesperetin regulates the inflammatory response caused by AMI in mouse models (Meng et al., 2018). Protein kinase A (PKA) inhibition enhances susceptibility to ferroptosis in NSCLC cells and could improve the efficacy of ferroptosis inhibitors in the treatment of NSCLC patients (Shan et al., 2023). The effects of these drugs on the two diseases warrant further investigation. Our results provide further ideas for drug therapy of the two diseases.

In summary, we comprehensively analyzed public databases and gene expression microarray data from NSCLC and AMI patients and healthy controls. We identified eight common signature genes (*CEBPA*, *TGFBR2*, *EZH2*, *JUNB*, *JUN*, *FOS*, *PLAU*, *COL1A1*) and their co-regulated pathways between NSCLC and AMI. All eight common signature genes have high diagnostic value, among which *EZH2* and *TGFBR2* perform well. In the validation datasets, *COL1A1*, *PLAU*, *JUNB*, *JUN*, *TGFBR2*, and *EZH2* retain their high diagnostic effect. Undoubtedly, detecting shared hub genes and pathways in NSCLC and AMI provides new insights into potential therapeutic targets for patients with both diseases and this finding may facilitate the diagnosis of LC-related MI. Among these genes, *COL1A1* may be involved in the occurrence and progression of the two diseases by participating in the EMT process and the formation of arterial dissection separately. Meanwhile, the association of *PLAU* with these two diseases may be related to tumor invasiveness and macrophage activity, respectively. We also constructed miRNA-hub and TF-hub networks, in which five miRNAs and 6 TFs were found to be active in the network. These miRNAs and TFs are implicated in various cellular processes, including cell proliferation, differentiation, apoptosis, and immune responses, which are critical to the development and progression of both NSCLC and AMI. Furthermore, we predicted potential drugs based on common targets and obtained ten candidate drugs, providing more treatment options for related diseases. It is worth emphasizing that zoledronic acid targets two hub genes simultaneously and has shown in past experiments that it can reduce the risk of both cancer and cardiovascular disease. Therefore, our research suggests that zoledronic acid anhydrous may have great therapeutic prospects as a treatment for the comorbidity of lung cancer and myocardial infarction.

However, this study’s limitations involve the need for further experimental validation of the shared hub genes and pathways identified in AMI and NSCLC. There is no specific relationship among the selected datasets. If patients with AMI who also have NSCLC were included as the research subjects, the results might be more representative. Additionally, the diagnosis of NSCLC and AMI should not rely solely on these signature genes and pathways but also needs to consider clinical symptoms and laboratory tests. Also, the availability of extensive and valuable public datasets for AMI and NSCLC is somewhat limited, highlighting the need for additional data in future studies to substantiate our findings.

## Data Availability

Publicly available datasets were analyzed in this study. This data can be found here: https://www.ncbi.nlm.nih.gov/geo/query/acc.cgi?acc=gse166780 https://www.ncbi.nlm.nih.gov/geo/query/acc.cgi?acc=GSE8569.
